# Ultrasound Entropy Imaging Based on the Kernel Density Estimation: A New Approach to Hepatic Steatosis Characterization

**DOI:** 10.3390/diagnostics13243646

**Published:** 2023-12-12

**Authors:** Ruiyang Gao, Po-Hsiang Tsui, Shuicai Wu, Dar-In Tai, Guangyu Bin, Zhuhuang Zhou

**Affiliations:** 1 Department of Biomedical Engineering, Faculty of Environment and Life, Beijing University of Technology, Beijing 100124, China; gry990416@163.com (R.G.); wushuicai@bjut.edu.cn (S.W.); 2 Department of Medical Imaging and Radiological Sciences, College of Medicine, Chang Gung University, Taoyuan 333323, Taiwan; tsuiph@mail.cgu.edu.tw; 3 Research Center for Radiation Medicine, Chang Gung University, Taoyuan 333323, Taiwan; 4 Division of Pediatric Gastroenterology, Department of Pediatrics, Chang Gung Memorial Hospital at Linkou, Taoyuan 333423, Taiwan; 5 Department of Gastroenterology and Hepatology, Chang Gung Memorial Hospital at Linkou, Chang Gung University, Taoyuan 333423, Taiwan; tai48978@cgmh.org.tw

**Keywords:** quantitative ultrasound, backscatter envelope statistics, ultrasound entropy imaging, kernel density estimation, probability density function, ultrasound tissue characterization, ultrasound backscattered signals, hepatic steatosis

## Abstract

In this paper, we present the kernel density estimation (KDE)-based parallelized ultrasound entropy imaging and apply it for hepatic steatosis characterization. A KDE technique was used to estimate the probability density function (PDF) of ultrasound backscattered signals. The estimated PDF was utilized to estimate the Shannon entropy to construct parametric images. In addition, the parallel computation technique was incorporated. Clinical experiments of hepatic steatosis were conducted to validate the feasibility of the proposed method. Seventy-two participants and 204 patients with different grades of hepatic steatosis were included. The experimental results show that the KDE-based entropy parameter correlates with log_10_ (hepatic fat fractions) measured by magnetic resonance spectroscopy in the 72 participants (Pearson’s *r* = 0.52, *p* < 0.0001), and its areas under the receiver operating characteristic curves for diagnosing hepatic steatosis grades ≥ mild, ≥moderate, and ≥severe are 0.65, 0.73, and 0.80, respectively, for the 204 patients. The proposed method overcomes the drawbacks of conventional histogram-based ultrasound entropy imaging, including limited dynamic ranges and histogram settings dependence, although the diagnostic performance is slightly worse than conventional histogram-based entropy imaging. The proposed KDE-based parallelized ultrasound entropy imaging technique may be used as a new ultrasound entropy imaging method for hepatic steatosis characterization.

## 1. Introduction

Nonalcoholic fatty liver disease (NAFLD) affects 25% of the global population and has become the most common cause of chronic liver disease in the world [1]. NAFLD has the updated names of metabolic dysfunction-associated fatty liver disease (MAFLD) [2] or metabolic dysfunction-associated steatotic liver disease (MASLD) [3]. Hepatic steatosis is a key manifestation of NAFLD, MAFLD, or MASLD. Currently, liver biopsy is still the golden standard for diagnosing hepatic steatosis. However, liver biopsy is invasive and may cause sampling errors and complications.

Ultrasound is a first-line diagnostic tool for the assessment and management of hepatic steatosis. However, the commonly used ultrasound B-mode imaging technique is qualitative and only uses the amplitude information of the envelopes of ultrasound backscattered signals. Quantitative ultrasound extracts quantified frequency, phase, or statistical information from ultrasound backscattered signals [4,5,6] and can be a complement to conventional ultrasound B-mode imaging.

Ultrasound backscatter envelope statistics parametric imaging is an important form of quantitative ultrasound [7,8,9]. These techniques can be classified into model-based and non-model-based ones. Typical model-based techniques are ultrasound Nakagami imaging [10,11,12,13] and homodyned-K imaging [14,15] that are based on the generalized statistical models of backscatter envelopes. Ultrasound entropy imaging uses the information-theoretic entropy of ultrasound backscatter envelopes, without a perquisite for satisfying a specific statistical model [9], which is of growing research interest in the field of quantitative ultrasound.

Currently, the Shannon entropy is the mostly studied entropic imaging method for ultrasound tissue characterization [9,11,16,17,18,19,20,21], as it is a well-suited method for quantifying ultrasound signal uncertainty or complexity. A histogram-based method is usually used to estimate the probability of gated ultrasound backscattered signals, which is then used to estimate the Shannon entropy [9]. Conventional histogram-based ultrasound entropy imaging has been used for the ultrasound characterization of different tissues [9], including a hepatic steatosis assessment [16]. The literature survey shows that ultrasound Shannon entropy imaging is the mostly used entropic imaging approach for characterizing biological tissues, and histogram-based methods are the dominant estimators for ultrasonic Shannon entropy [9,11,16,17,18,19,20,21]. However, conventional histogram-based ultrasound entropy imaging is affected by histogram settings, such as the number of histogram bins, and has a limited dynamic range, as demonstrated in our previous work [16].

In this paper, we propose the kernel density estimation (KDE)-based ultrasound Shannon entropy imaging technique to overcome the drawbacks of conventional histogram-based ultrasound Shannon entropy imaging and apply it to a hepatic steatosis assessment. A KDE technique is used to estimate the probability density function (PDF) of gated ultrasound backscattered signals. The estimated PDF is then used to estimate the Shannon entropy to construct parametric images. In addition, we incorporated the parallel computation technique we recently introduced for ultrasound homodyned-K imaging [22] into the KDE-based ultrasound entropy imaging. The clinical data for hepatic steatosis are used to validate the feasibility of the proposed KDE-based parallelized ultrasound entropy imaging method. The major contributions of this study are as follows.

(1) To the best of our knowledge, this study is the first to propose KDE-based ultrasound Shannon entropy imaging, which overcomes the drawbacks of conventional histogram-based ultrasound Shannon entropy imaging methods.
(2) Parallelized computation techniques are incorporated to speed up the proposed algorithm.
(3) Clinical validations of hepatic steatosis evaluation are performed.

## 2. Materials and Methods

### 2.1. KDE-Based Shannon Entropy Estimation of Ultrasound Backscattered Signals

For a variable, *X*, the information-theoretic Shannon entropy, *E*, is defined as:

(1) $$E(X)=-\sum_{i=1}^{n}p(x_{i})\log_{2}[p(x_{i})],$$

where *p*(*x_i_*) denotes the probability that *X* is in the state *x_i_*, *n* is the number of states, and $p(x_{i})\log_{2}[p(x_{i})]$ is defined as 0 if *p*(*x_i_*) = 0.

In the context of ultrasound backscattered signals, the information-theoretic entropy is a quantitative measure of signal uncertainty or complexity. The Shannon entropy of ultrasound backscattered signals is usually estimated by Equation (1), with statistical histograms used for the probability estimation [16]. However, the histogram-based entropy estimate of the ultrasound backscattered signals, denoted by *E*_hist_, was affected by histogram settings, such as the number of histogram bins [16]. Furthermore, *E*_hist_ was found to have limited dynamic ranges in previous ultrasound entropy imaging studies [16].

To overcome the shortcomings of the histogram-based entropy estimation, we considered non-parametric KDE techniques to estimate the PDF of ultrasound backscattered signals and used the estimated PDF to estimate the entropy. For a continuous random variable, *Y*, with a PDF *f*(*y*), the Shannon entropy is defined as [23,24]:

(2) $$E(Y)=-\int_{-\infty }^{\infty }f(y)\ln f(y)dy\equiv -M[\ln f(y)],$$

where *M*[.] is the statistical mean operation. Then, for a discrete random variable, *Z*, with *N* samples, the Shannon entropy can be estimated by [24,25]:

(3) $$E(Z)=-M[\ln f(z)]=-\frac{1}{N}\sum_{i=1}^{N}\ln f(Z_{i}),$$

where *f*(*z*) is the PDF estimated by the KDE methods [25]:

(4) $$f(z)=\frac{1}{Nh}\sum_{i=1}^{N}K\left(\frac{z-Z_{i}}{h}\right),$$

where *h* is the bandwidth parameter and *K*(.) is a kernel function. In this study, we used the Gaussian kernel function:

(5) $$K(v)=\exp(-v^{2}/2)/\sqrt{2\pi }.$$

The reason why we selected the Gaussian kernel function is discussed in the Section 4. The KDE-based entropy estimate of the ultrasound backscattered signals, denoted by *E*_KDE_, was obtained by Equations (3)–(5):

In this study, the bandwidth parameter, *h*, was calculated adaptively, based on a one-dimensional signal, *S*, using Scott’s method [26]:

(6) $$h=\beta {\left[\frac{4}{N(d+2)}\right]}^{\frac{1}{d+4}},$$

where *N* is the length of the signal, *S*; *d* is the dimension of *S*, *d* = 1; and *β* is a coefficient that is adaptively calculated by the mean absolute deviation of *S*:

(7) β=E[|S−E[S]|]

where *E*[.] is the expectation operator. It can be seen that the bandwidth parameter, *h*, is a function of the input signal, *S*. In this work, *h* did not need to be set specifically; instead, it was automatically calculated based on *S*.

### 2.2. KDE-Based Parallelized Ultrasound Shannon Entropy Imaging Method

The parametric imaging of KDE-based entropy estimates of ultrasound backscattered signals, i.e., *E*_KDE_ parametric imaging, was proposed in this study. In addition, we incorporated the parallel computation technique proposed in our previous work [22] for the algorithmic acceleration of ultrasound homodyned-K imaging.

**Figure 1:**
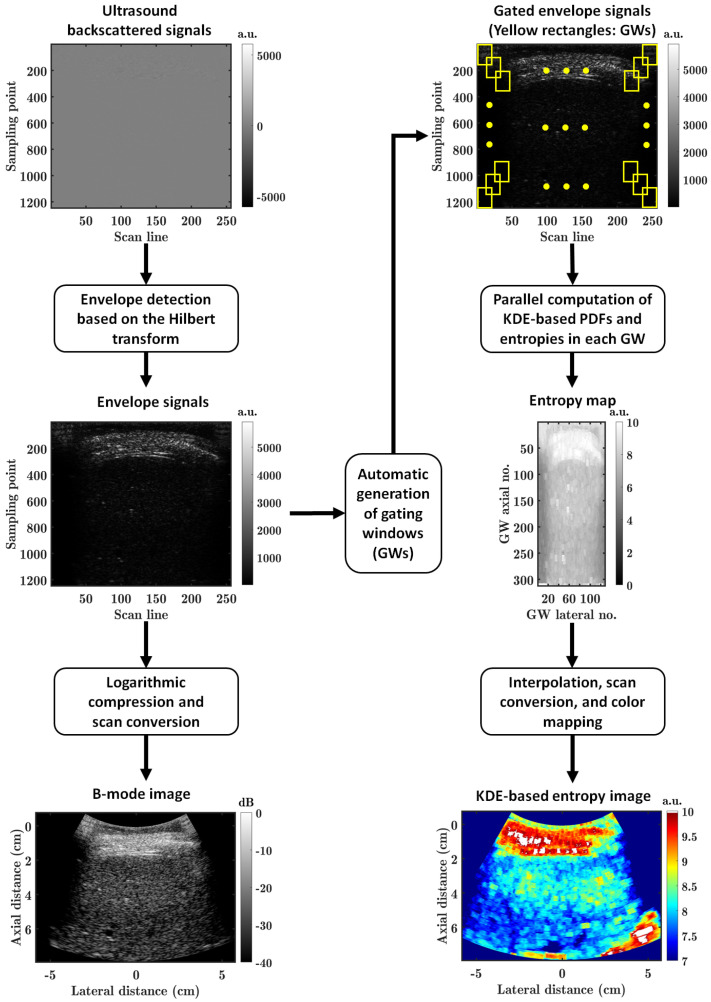
Flowchart of the proposed kernel density estimation (KDE)-based parallelized ultrasound entropy imaging method. GW: gating window; PDF: probability density function; a.u.: arbitrary unit; no.: number.

Figure 1 shows the flowchart of the KDE-based parallelized ultrasound entropy imaging method proposed in this work. For an input frame of ultrasound backscattered signals, a frame of envelope signals was obtained by envelope detection based on the Hilbert transform. Then, logarithmic compression and scan conversion were performed on the frame of envelope signals to obtain an ultrasound B-mode image.

For quantitative ultrasound imaging, a sliding window technique was frequently used in the previous work [16], where a gating window was slid across the entire frame of envelope signals, with an overlap ratio (lateral × axial) between two adjacent gating windows. The quantitative ultrasound parameters were then estimated for each gating window, respectively. This generally required two nested loops in the algorithmic implementation, which was computationally inefficient, especially when the computational complexity of the quantitative ultrasound parameter estimation was high.

In this work, we introduced, for the first time, a parallel computation [22] into the ultrasound entropy imaging method to improve the time efficiency. Firstly, a series of gating windows were automatically generated in the frame of envelope signals, indicated by the yellow rectangles in Figure 1. Secondly, a parallel computation of KDE-based PDFs and entropies was performed on the gated ultrasound backscattered signals in each gating window, and an entropy map was created. Each pixel of the entropy map corresponded to a KDE-based entropy estimate, *E*_KDE_, in a gating window. Finally, two-dimensional (2D) image interpolation, scan conversion, and color mapping were conducted on the entropy map to obtain the KDE-based ultrasound entropy image. The 2D image interpolation was performed because the entropy map had a size smaller than the size of the original backscattered RF signals (Figure 1), and the entropy map was resized to the same size of the original RF signals using an interpolation algorithm.

### 2.3. Clinical Validations

To validate the feasibility of the proposed KDE-based parallelized ultrasound entropy imaging method, the ultrasound backscattered radiofrequency (RF) signals of hepatic steatosis used in our previous work [27] were included. This study was approved by the Institutional Review Board of Chang Gung Memorial Hospital in Taiwan to revisit the clinical data for the signal analysis. Informed consent was obtained from all the participants. The RF signals included two groups. In group I, the RF signals were acquired from 72 liver donors, with the hepatic fat fraction (HFF) measured by magnetic resonance spectroscopy (MRS) as the reference standard of the hepatic steatosis evaluation. Group II included 204 patients with chronic hepatitis B, for which liver biopsy was used as the reference standard. For these patients, the liver specimen was obtained from the right liver lobe through an intercostal approach with the guidance of ultrasound imaging. All the specimens were placed in formalin and sent to the Department of Pathology, Chang Gung Memorial Hospital, Linkou, Taiwan, for a histological examination. The samples were fixed in paraffin and stained with hematoxylin–eosin, which were read on-site by expert liver pathologists. Those samples having a minimum of six portal tracts were considered suitable for a histologic evaluation [28]. According to Brunt et al. [29], hepatic steatosis grades were classified into G0 (steatosis involving < 5% of hepatocytes), G1 (steatosis involving 5%–32.99% of hepatocytes), G2 (steatosis involving 33%–66% of hepatocytes), and G3 (steatosis involving > 66% of hepatocytes), corresponding to normal liver, mild steatosis, moderate steatosis, and severe steatosis, respectively. The RF signals were determined with an ultrasound scanner (Model 3000, Terason, Burlington, MA, USA) with a convex-array transducer (Model 5C2A, Terason). The transducer had a central frequency of 3 MHz and a pulse length (PL) of ~2.3 mm. The sampling frequency of the ultrasound imaging system was 12 MHz.

### 2.4. Clinical Ultrasound Backscattered Signal Processing Method

For an input frame of the ultrasound backscattered RF signals, envelope signals were detected by using the Hilbert transform. The detected envelope signals were utilized to construct ultrasound B-mode and KDE-based entropy (i.e., *E*_KDE_) images (Figure 1). The gating window was sized to 1 PL × 1 PL, i.e., 2.3 mm × 2.3 mm (lateral × axial), as the ultrasound entropy imaging method supported a small gating window [16]. The dynamic range of the ultrasound B-mode imaging was set to 40 dB. The overlap ratio was set to 90% × 90% (lateral × axial) [22], as the parallel computation technique we incorporated supported a higher parametric imaging resolution and a faster computation result at the same time. For the sake of comparison, the conventional histogram-based entropy (i.e., *E*_hist_) imaging was analyzed with the same procedure and settings as *E*_KDE_ parametric imaging, except that the entropy was estimated using a histogram-based method and that a different color mapping technique was used as the dynamic range was lower. The number of histogram bins of 40 was used for *E*_hist_ parametric imaging [16]. A quantitative evaluation was performed by manually delineating a liver region of interest (ROI) on an ultrasound B-mode image [27]. The delineated ROI was applied to the *E*_hist_ and *E*_KDE_ parametric images corresponding to the B-mode image to calculate the mean parametric image pixel values for the ROI. The dynamic range, *DR*, of the *E*_hist_ and *E*_KDE_ estimates for the liver ROI was calculated by *DR* = *E*_max_ − *E*_min_, where *E*_max_ and *E*_min_ were the maximum and minimum values of the entropy estimates for the liver ROI, respectively. All the signal processing procedures were performed using MATLAB software (version 2020a, The MathWorks, Natick, MA, USA). The MATLAB subroutine Hilbert() was used for the envelope detection of ultrasound backscattered signals. The MATLAB subroutine histcounts() was utilized for the histogram-based probability estimation of ultrasound backscattered signals. The MATLAB subroutine ksdensity() was utilized for the KDE-based PDF estimation of the ultrasound backscattered signals.

The MATLAB subroutine blockproc() was used for the parallel computation of the ultrasound entropy images. A frame of uncompressed envelope signals, *ENV*s, was automatically divided into several blocks. The number of blocks was determined by the block size (BS) in the axial and lateral directions, BS_axial_ × BS_lateral_, and by the block overlap (BO) in the axial and lateral directions, BO_axial_ × BO_lateral_. Then, the parallelized computation of the entropy parameter matrix, *ENT*, in all the blocks was conducted using the subroutine blockproc(), i.e., *ENT* = blockproc(*ENV*, [*m*, *n*], @(block_struct) blockEnt(block_struct), ‘BorderSize’, [*v*, *w*], ‘UseParallel’, true, ‘TrimBorder’, false), where *v* = floor(BS_axial_ * BO_axial_/2); *w* = floor(BS_lateral_ * BO_lateral_/2); *m* = BSaxial − 2*v; *n* = BSlateral − 2*w; block_struct is a structure defined in MATLAB; and blockEnt() is a callback function for estimating the entropy parameter of one block using the KDE estimator or conventional histogram-based entropy estimator. It can be seen that parallelization and the communication between parallelized blocks were automatically conducted by the blockproc() subroutine. The blockproc() subroutine extracted each block from the envelope signals, *ENV*s, passed it to the callback function blockEnt(), and assembled the returned blocks to obtain the matrix of the estimated values of the entropy parameters (i.e., *ENT*).

### 2.5. Statistical Analysis

For group I, the Pearson’s correlation coefficient, *r*, between the mean entropy (*E*_hist_ and *E*_KDE_) values of the ROIs and log_10_(HFF) was computed, where log_10_(HFF) was used rather than the HFF because our previous studies demonstrated that quantitative ultrasound parameters had a better correlation with log_10_(HFF) than with HFF [16,27]. For group II, a receiver operating characteristic (ROC) analysis was conducted and the area under the ROC curve (AUC) was calculated using the 95% confidence interval (CI), in order to evaluate the diagnostic performances of *E*_hist_ and *E*_KDE_ parametric imaging for classifying different grades of hepatic steatosis. We conducted three binary classifications: G0 versus G1–G3 (≥G1), G0–G1 versus G2–G3 (≥G2), and G0–G2 versus G3 (≥G3). In addition, a box plot was created. All the statistical analyses were performed using MATLAB software (version 2020a, The MathWorks, Natick, MA, USA).

## 3. Results

**Figure 2:**
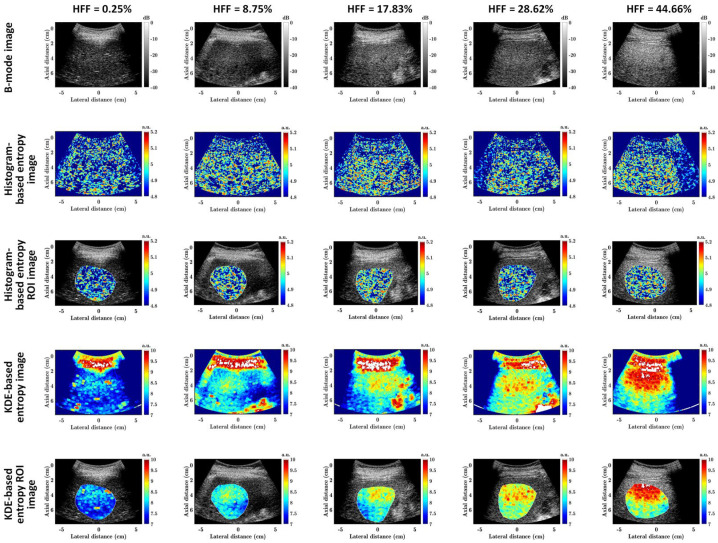
Representative ultrasound B-mode (first row) as well as *E*_hist_ (second row) and *E*_KDE_ (the fourth row) parametric images and their liver ROI images (third and fifth rows) corresponding to different HFFs in group I. The first to fifth columns correspond to HFFs = 0.25%, 8.75%, 17.83%, 28.62%, and 44.66%, respectively. HFF: hepatic fat fraction; ROI: region of interest; a.u.: arbitrary unit.

Figure 2 shows the representative ultrasound B-mode as well as *E*_hist_ and *E*_KDE_ parametric images and their liver ROI images corresponding to different HFFs in group I. With the increase in the HFF, the B-mode image shows a generally increasing trend in image brightness, and the shadings of *E*_hist_ and *E*_KDE_ parametric images also show an increasing trend, especially for the liver ROIs. However, the B-mode imaging was qualitative, and *E*_hist_ parametric imaging had a lower dynamic range and a lower image contrast than *E*_KDE_ parametric imaging.

**Figure 3:**
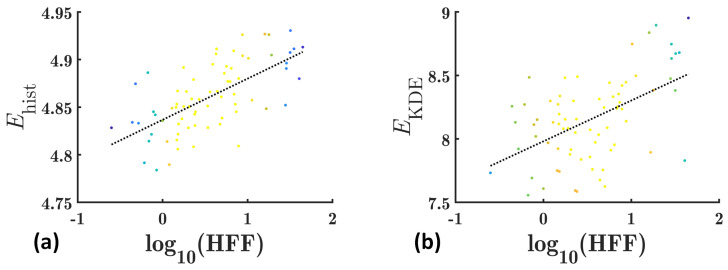
Scatter plots of the mean *E*_hist_ (**a**) and *E*_KDE_ (**b**) values for the liver ROIs as a function of log_10_(HFF) in group I. The dotted lines are fitting lines. HFF: hepatic fat fraction; ROI: region of interest.

**Figure 4:**
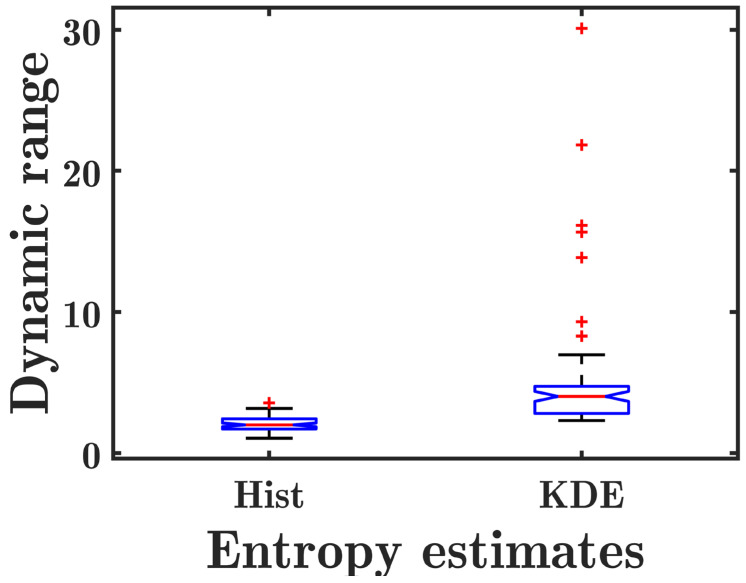
Box plots of the dynamic ranges of the conventional histogram-based entropy *E*_hist_ (Hist) and the proposed KDE-based entropy *E*_KDE_ (KDE) estimates for the liver ROIs. The dynamic ranges of *E*_KDE_ are significantly higher than those of *E*_hist_ (*p* < 0.0001 with a paired *t*-test).

Figure 3 shows the scatter plots of the mean *E*_hist_ and *E*_KDE_ values for the liver ROIs as a function of log_10_(HFF) in group I. Both *E*_hist_ and *E*_KDE_ were correlated with log_10_(HFF). However, *E*_hist_ had a higher correlation coefficient (*r* = 0.65, *p* < 0.0001) with log_10_(HFF) than *E*_KDE_ (*r* = 0.52, *p* < 0.0001). It is shown that the mean value of *E*_hist_ in group I ranges from ~4.75 to ~4.95, while that of *E*_KDE_ ranges from ~7.5 to ~9.0, indicating that the dynamic range of *E*_KDE_ parametric imaging is much higher than that of *E*_hist_ parametric imaging. The box plots of the dynamic ranges of *E*_hist_ and *E*_KDE_ for the liver ROIs are shown in Figure 4. The dynamic range of *E*_KDE_ is significantly higher than that of *E*_hist_ (*p* < 0.0001).

**Figure 5:**
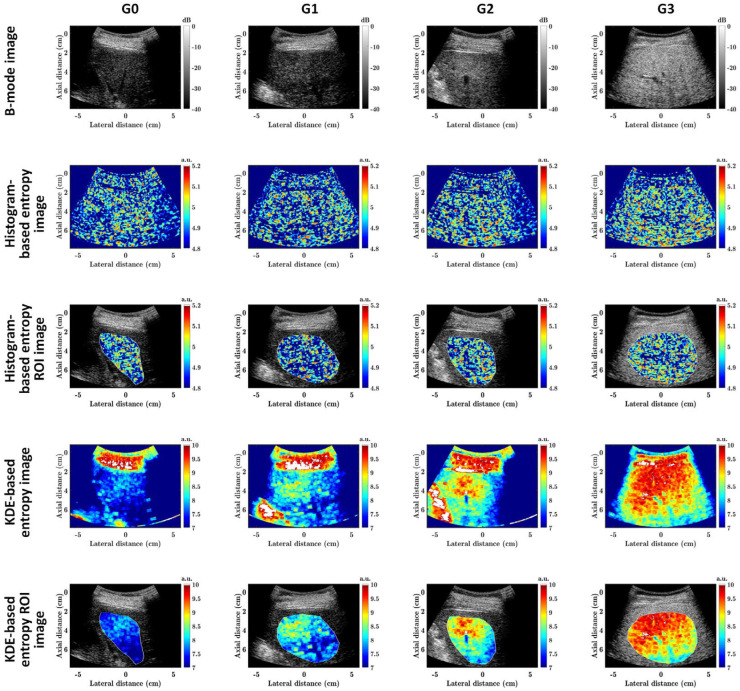
Representative ultrasound B-mode (first row) as well as *E*_hist_ (second row) and *E*_KDE_ (fourth row) parametric images and their liver ROI images (third and fifth rows) corresponding to different hepatic steatosis grades in group II. The first to fourth columns correspond to hepatic steatosis grades = G0, G1, G2, and G3, respectively. ROI: region of interest; a.u.: arbitrary unit.

For group II, liver biopsy examinations revealed that the numbers for G0, G1, G2, and G3 were 80, 70, 36, and 18, respectively. Figure 5 shows representative ultrasound B-mode as well as *E*_hist_ and *E*_KDE_ parametric images and their liver ROI images corresponding to different hepatic steatosis grades in group II. As the hepatic steatosis grade increases from G0 to G3, the B-mode image brightness and the entropy image shadings generally increase, similar to those in group I. Both *E*_hist_ and *E*_KDE_ parametric imaging were capable to visually characterize different grades of hepatic steatosis.

**Figure 6:**
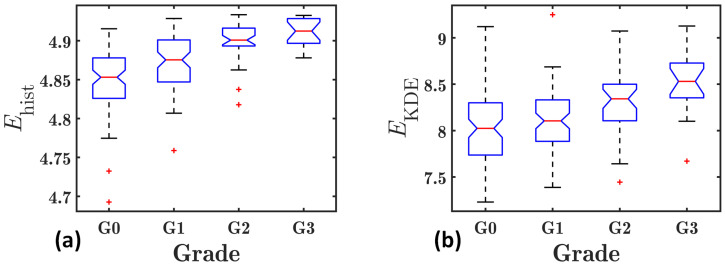
Box plots of the mean *E*_hist_ (**a**) and *E*_KDE_ (**b**) values for the liver ROIs corresponding to each hepatic steatosis grade in group II.

Figure 6 shows the box plots of the mean *E*_hist_ and *E*_KDE_ values of the liver ROIs corresponding to each hepatic steatosis grade in group II. Both *E*_hist_ and *E*_KDE_ increased with the increases in hepatic steatosis grades, indicating they were capable of detecting the different hepatic steatosis grades. Furthermore, Figure 5 and Figure 6 show that *E*_KDE_ parametric imaging presents a much higher dynamic range in group II than *E*_hist_ parametric imaging.

**Figure 7:**
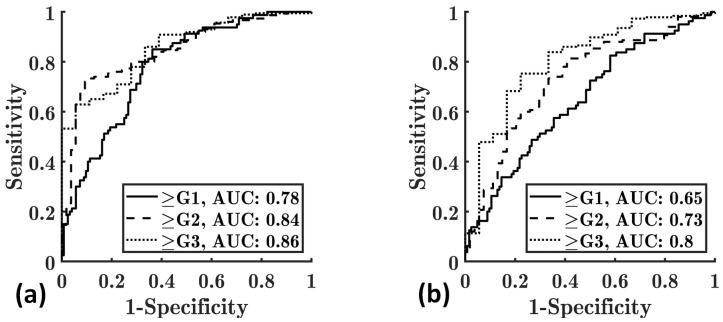
ROC curves for diagnosing different grades of hepatic steatosis using *E*_hist_ (**a**) and *E*_KDE_ (**b**) parametric imaging methods in group II. ROC: receiver operating characteristic; AUC: area under the ROC curve.

Figure 7 shows the ROC curves for diagnosing different grades of hepatic steatosis using *E*_hist_ and *E*_KDE_ parametric imaging methods in group II. The AUCs for diagnosing hepatic steatosis grades ≥ G1, ≥G2, and ≥G3 using the *E*_hist_ parameter were 0.78, 0.84, and 0.86, respectively, and those using the *E*_KDE_ parameter were 0.65, 0.73, and 0.80, respectively. Both *E*_hist_ and *E*_KDE_ parametric imaging methods were capable of diagnosing different hepatic steatosis grades. However, *E*_hist_ parametric imaging had higher AUCs than *E*_KDE_ parametric imaging. Table 1 summarizes the performance comparisons of *E*_hist_ and *E*_KDE_ parametric imaging methods for diagnosing different grades of hepatic steatosis in group II.

## 4. Discussion

In this study, we proposed KDE-based ultrasound entropy imaging for characterizing clinical hepatic steatosis. In addition, we incorporated the parallel computation technique we recently introduced [22] into ultrasound entropy imaging. To the best of our knowledge, the proposed KDE-based parallelized ultrasound entropy imaging technique is the first to apply non-parametric entropy estimation and parallel computation techniques to ultrasound entropy imaging. Note that the parallel computation technique [22] can be easily extended to other quantitative ultrasound applications.

Conventional histogram-based ultrasound entropy imaging is based on probability estimations from ultrasound backscattered signals by using statistical histograms, while the proposed KDE-based ultrasound entropy imaging was built by using PDF estimations from ultrasound backscattered signals by using KDE. The former is known as a parametric estimation, and the latter a non-parametric estimation. Compared to conventional histogram-based ultrasound entropy imaging, the proposed KDE-based ultrasound entropy imaging has a much greater imaging dynamic range (which can be observed in Figure 2, Figure 3, Figure 4, Figure 5 and Figure 6), although its correlation coefficients with log_10_(HFF) and diagnostic performance (AUC) are lower. Moreover, histogram-based ultrasound entropy imaging is affected by histogram settings, such as the histogram bin number [16]. Specifically, when the number of histogram bins or histogram limits alter, the probability values change, and the entropy estimate varies correspondingly, making the histogram-based entropy method a relative quantitative ultrasound parameter. These two drawbacks of histogram-based ultrasound entropy imaging are well addressed by the proposed KDE-based ultrasound entropy imaging.

Acoustically, a biological tissue can be modeled as an ensemble of small particles that scatter ultrasound waves, namely, acoustic scatterers. In the context of the liver, the effective acoustic scatterers of normal liver tissue may be considered as liver lobules, which are regularly or quasi-regularly distributed. In human hepatic steatosis, large fatty droplets usually fill hepatocytes and displace nuclei to the periphery [30]. A simplified acoustic model of human hepatic steatosis can be created on the basis of a scattering medium with randomly distributed acoustic scatterers due to fat-infiltrated hepatocytes from large droplet steatosis in addition to a large number of effective acoustic scatterers (i.e., liver lobules). Under this condition, increasing the severity of hepatic steatosis corresponds to an increase in the uncertainty or complexity of ultrasound backscattered signals, which is well characterized by the Shannon entropy [31].

The reason why we chose the Gaussian kernel for KDE-based ultrasound entropy imaging is discussed as below. Two kernel functions were recommended by Smolíková et al. [24] for the PDF estimation of ultrasound backscattered signals, i.e., the Gaussian and Epanechnikov kernels, and they argued that the Epanechnikov kernel has a slightly higher efficiency than the Gaussian kernel. From our experiments, we found that the Gaussian kernel yielded a slightly better diagnostic performance than the Epanechnikov kernel, and the Gaussian kernel produced a higher entropy imaging contrast than the Epanechnikov kernel. Another kernel function that may be suitable for estimating the PDF from ultrasound backscattered signals is the Gamma kernel [32,33]. We implemented KDE-based ultrasound entropy imaging with the Gamma kernel; however, we found its computational speed was very low, even with the incorporation of the parallel computation technique [22]. Therefore, we selected the Gaussian kernel when estimating the PDF of ultrasound backscattered signals.

In this paper, the Shannon entropy was estimated and imaged using the PDF estimated by KDE techniques. However, it should be noted that the proposed method can be easily extended to other entropy measures, such as the Renyi and Tsallis entropy methods, which can be estimated by using the KED-based PDF. Note that there are other kinds of entropy measures in addition to the Shannon, Renyi, and Tsallis entropy methods. In our previous work [34], we introduced ultrasound sample entropy imaging for liver tissue characterizations. Unlike the Shannon entropy based on probability or PDF estimations by conventional histogram-based or proposed KDE-based methods, sample entropy was determined by the dimension parameter, *m*, and the tolerance parameter, *r* [34]. The drawback of the sample entropy method lies in the fact that the two critical parameters, *m* and *r*, should be set empirically or experimentally. This drawback is similar to the drawback of conventional histogram-based Shannon entropy estimates, where the number of histogram bins and limits should be set specifically. In contrast, the proposed KDE-based Shannon entropy estimation method is an adaptive method, with the key parameter, the bandwidth parameter, *h*, adaptively determined from the test signal.

We presented a new ultrasound Shannon entropy estimation and imaging method. This method overcomes conventional histogram-based Shannon entropy estimation and imaging methods in that it is based on the PDF estimation by KDE techniques, and it is an adaptive method that is not affected by parameter settings, such as histogram bins and limits, yielding much higher imaging dynamic range and contrast results than conventional histogram-based methods. Furthermore, we incorporated parallelized computation techniques into the proposed KDE-based ultrasound Shannon entropy imaging method, which achieved a higher entropic parametric imaging resolution and a higher computation speed at the same time. The proposed method may serve as a new quantitative ultrasound method for tissue characterization. Although we used a hepatic steatosis evaluation for the clinical validations, the proposed method could be easily extended to other applications of ultrasound tissue characterizations for the sake of disease diagnosis, therapy monitoring, and prognosis prediction, as the proposed method was related to alterations in tissue microstructures, i.e., acoustic scatterers. In addition, the proposed method can be conveniently integrated into an ultrasound scanner and is compatible with existing ultrasound imaging systems, serving as a complement to conventional B-mode ultrasound imaging.

This study presented limitations. The proposed KDE-based entropy imaging method was only validated for ultrasound backscattered signals collected from one ultrasound scanner platform. Because we did not eliminate the system-dependent factors, such as the diffraction effect, the entropy estimate could be varied when using a different ultrasound scanner. The diagnostic performance of the proposed KDE-based entropy imaging was worse than the conventional histogram-based entropy imaging method. In future work, the performance of KDE-based entropy imaging when diagnosing different hepatic steatosis grades can be validated by more ultrasound scanners. The performance for diagnosing steatosis grade ≥ G1 can be improved, and the radiomics features can be extracted from the KDE-based entropy images to enhance the classification performance. In addition, system-dependent effects can be alleviated using methods, such as the reference phantom method [35].

## 5. Conclusions

In this paper, we proposed KDE-based parallelized ultrasound entropy imaging and applied it for hepatic steatosis characterization. The proposed method overcame the limitations of conventional histogram-based ultrasound entropy imaging methods, including limited dynamic ranges and histogram settings dependence. Clinical experiments were conducted to validate the feasibility of the proposed KDE-base parallelized ultrasound entropy imaging method. The proposed KDE-based parallelized ultrasound entropy imaging method can be used as a new ultrasound entropy imaging method for hepatic steatosis characterization.

## Figures and Tables

**Table 1:** *E*_hist_ and *E*_KDE_ parametric imaging for diagnosing different grades of hepatic steatosis in group II.

Parameter	≥G1		≥G2		≥G3	
	*E*_hist_	*E*_KDE_	*E*_hist_	*E*_KDE_	*E*_hist_	*E*_KDE_
Cutoff value	4.88	8.10	4.89	8.30	4.90	8.35
Youden’s Index	0.48	0.22	0.62	0.40	0.50	0.53
Sensitivity (%)	80.00	57.50	73.33	73.33	78.00	75.27
Specificity (%)	67.74	64.52	88.89	66.67	72.22	77.78
LR+	2.48	1.62	6.60	2.20	2.81	3.39
LR-	0.30	0.66	0.30	0.40	0.31	0.32
PPV (%)	61.54	51.11	94.83	85.94	96.67	97.22
NPV (%)	84.00	70.18	54.55	47.37	24.07	23.33
AUC, 95% CI	0.71–0.85	0.57–0.73	0.79–0.90	0.66–0.80	0.79–0.92	0.72–0.89
AUC	0.78	0.65	0.84	0.73	0.86	0.80

*Note:* LR+: positive likelihood ratio; LR-: negative likelihood ratio; PPV: positive predictive value; NPV: negative predictive value; AUC: area under the receiver operating characteristic curve; CI: confidence interval.

## Data Availability

The ultrasound radiofrequency data and the ROI masks may be provided upon reasonable requests for scientific research purposes.
